# Can cornelian cherry mask bitter taste of probiotic chocolate? Human TAS2R receptors and a sensory study with comprehensive characterisation of new functional product

**DOI:** 10.1371/journal.pone.0243871

**Published:** 2021-02-08

**Authors:** Oskar Szczepaniak, Maria Jokiel, Kinga Stuper-Szablewska, Daria Szymanowska, Marcin Dziedziński, Joanna Kobus-Cisowska

**Affiliations:** 1 Department of Gastronomy Sciences and Functional Foods, Poznań University of Life Sciences, Poznań, Poland; 2 PORT Polish Center for Technology Development, Wrocław, Poland; 3 Department of Chemistry, Poznań University of Life Sciences, Poznań, Poland; 4 Department of Biotechnology and Food Microbiology, Poznań University of Life Sciences, Poznań, Poland; Institute for Biological Research, SERBIA

## Abstract

Cornelian cherry (*Cornus mas* L.) fruits are a valuable source of bioactive compounds that are responsible for the perception of bitter taste of chocolate products. The aim of the study was to validate the inhibitory effect of *Cornus mas* on the TAS2R3 and TAS2R13 bitter taste receptors and to assess the effect of masking the bitter taste of dark chocolate with the help of the sensory panel. Dark chocolate was prepared with an addition of 5% of freeze-dried cornelian cherry fruits and 10^8^ CFU/g of *Bacillus coagulans* probiotic strains. Effect on the TAS2R receptors was evaluated in specially transfected HEK293T cells, and the inhibition ratio was measured using the calcium release test. Moreover, the total polyphenol content, antioxidant activity and simulated intestinal *in vitro* digestion were determined for the samples. The tested chocolate products were rich in chlorogenic, caffeic and sinapic acids. The addition of cornelian cherry positively affected the antioxidant activity. The phytochemicals of *Cornus mas* decreased the TAS2R13 activity by 132% after a 2-minute interaction and, % at the same time, inhibited the TAS2R3 activity by 11.5. Meanwhile, chocolate with the addition of fruit was less bitter according to the sensory panel.

## Introduction

Cornelian cherry (*Cornus mas* L.) is a plant growing in Central and Eastern Europe. Its reddish oval fruits have been used for fruit preserves. Fruits of *C*. *mas* are rich in phenolic acids, flavonoids, iridoids, and anthocyanins, which increase the antioxidant and anti-proliferative effect of the first substance [1]. Currently, *C*. *mas* fruits are used in innovative functional products, i.a. ice cream, burgers and vinegar [2–4]. Apart from the large anti-inflammatory and antioxidative potential, the addition of cornelian cherry fruits also results in the modulation of taste. Recent studies have shown that the addition of the fruit may lead to either higher overall intensity of flavour or the perception of more intense sweetness [5, 6]. However, no effect has been studied on bitterness of final product with addition of cornelian, despite the fact that the raw fruits are sour and bitter.

The modulation of bitter taste seems to be important in the production of dark chocolate. The general tendency to avoid sugar forced food producers to add natural or synthetic sweeteners and search for molecular inhibitors of bitter taste receptors belonging to the TAS2R family [7–9]. It has been proven that natural flavonols may inhibit the perception of bitter taste in chocolate [10].

Due to the fact that functional foods are highly desired consumer goods and the consumption of confectionary should be limited, polyols, instead of sugar, were addeded to the tested chocolate products, which were also enriched with lyophilised cornelian cherry fruits and *Bacillus coagulans* strain. The key objective of the study was to assess the overall impact of cornelian cherry fruits on the perception of bitter taste and the activity of sweet taste receptors.

The second aim of our study was to check whether adding powdered cornelian cherry fruits to dark chocolate could mask the bitter flavour and enhance the perception of sweetness. It was also planned to check whether probiotic bacteria–*Bacillus coagulans* would be stable in the presence of chocolate and *C*. *mas* phenolic compounds after the simulated *in vitro* digestion process and could enhance functional properties of chocolate.

## Materials and methods

### Raw materials

Dark chocolate with a cocoa content of 55%, manufactured by the "Bars" Company (Włoszakowice, Poland), was used to prepare new chocolate samples [11]. In the study, cornelian cherry fruits, cv. *Bolestraszycki* collected in September 2018 in "Szynsad" plant in Dąbrówka Nowa (Błędów, Polska, 51°47’01’’N 20°43’04’’E), were used. They were stored at a temperature of -21°C until they were subjected to the freeze-drying process. *Bacillus coagulans* GBI-30, 6086, was used as a probiotic strain in the study.

### Fruit preparation

Frozen cornelian cherry fruits were dried in a freeze dryer (Christ, Germany) for 72 h, at a pressure of 1.030 bar and at the condensation temperature of -52°C. The temperature of the freeze dryer shelf was 24°C. Dried fruits with a moisture content not higher than 4% (±0.2%) were drilled and then ground to powder in a mill (GM-200, Retsch, Germany). The grinding process was carried out for 30 s at the rotational speed of blades of 7000 rpm. As a result, two fractions of particles, including 70% ranging from 0.4 to 0.8 mm and 30% ranging from 0.2 to 0.4 mm, were obtained.

### Chocolate preparation

Chocolate was melted in a stainless steel heater equipped with a stirrer with a rotation speed of 100 rpm and with a water mantel maintained at a temperature of 70°C. Freeze-dried cornelian cherry fruits were added in powdered form at a ratio of 5% (w/w) to the chocolate mass. The lyophilisate of *Bacillus coagulans* bacteria was added at a concentration of 10^8^ CFU/g of chocolate, melting in 1/5 of the final chocolate mass volume, and then refilling with chocolate with other additives. Chocolate mass was heated and stirred for 5 minutes, and then the forms with a dimension of 1cm x 1cm x 0.4 cm were filled and kept at 21°C until the mass solidified. Chocolate samples were stored at ambient temperature until the scheduled analysis was conducted. In the study, two variants of chocolate were prepared: one with 5% (w/w) of cornelian cherry fruits (CHF) and the other with 5% (w/w) of cornelian cherry fruits and *B*. *coagulans* (CHFB). Dark chocolate containing 55% of cocoa (CHC) was used as a control sample. The CHC sample contained the following ingredients: cocoa mass 42%, maltitol 41.9%, cocoa butter 13.4%, emulsifier: soy lecithin 2.7%.

### Extract preparation

As much as 20 g of chocolate was weighed in a conical flask with an accuracy of 0.01 g. Then, 100 mL of distilled water at ambient temperature was added to the flask. The maceration process was carried out for 1 hour at a temperature of 40°C. The extract was transferred to 50 mL centrifuge tubes and the lipid fraction was separated at 1500 rpm after 15 minutes (Thermofisher, UK). Then, the aqueous fraction was collected.

The cornelian cherry extract (BW) was prepared according to the authors’ own method [12], by macerating fruits in distilled water at a ratio of 1:5 (w/v), at a temperature of 40°C, for 30 minutes. Then, the extract was filtered through a filter paper (Alfachem, Poland) and stored at -21°C until further analysis.

### Effect of cornelian cherry on receptors

The aqueous extract of cornelian cherry fruits–BW and the extract diluted 10-fold with PBS (Biowest, USA) buffer (BWX) were used in the study.

#### Transfection with TAS2R13 and TAS2R3 genes

The fragment of the gene coding the chimeric gustducine-acting protein was amplified at the initial stage of the experiment. The synthetic sequence, coding G16-gust44 (IDT, Belgium), was used as a matrix for the amplification process. Then, the amplified gene was inserted into the pcDNA 3.1 vector (Invitrogen, United States). After checking the correctness of the sequence in terms of sequencing, the second stage of the study started, i.e. the transfection of HEK293T cells. The cells were transfected using the lipofection method. The protein expression was confirmed on the basis of the Western Blot of proteins present in cell lysates. The G16-gust44 protein has an atomic mass of 45 kDa.

Cells, the lysates of which gave the most intense signals in the Western-Blot analysis, were chosen for further analysis. Cell cultures were dissolved in DMEM medium with a high glucose content (Biowest, United States), supplemented with Glutamax (Gibco, United States), PenStrep (Gibco), FBS (VWR, Poland), and sodium pyruvate (Gibco) to obtain statistically one cell per well in a 96-well microplate. The microplate containing cell solutions prepared in this way had been incubated for 3 weeks until the colony grew from a single cell. The cell line obtained was transfected using the lipofection method by means of plasmids containing genes coding bitter taste receptors (TAS2R13 and TAS2R3). The plasmid vectors were purchased from GenScript (United States). The overexpression of TAS2R13 and TAS2R3 was confirmed on the basis of the Western Blot test.

Cell lysates were separated using electrophoresis with the use of 10% poliacrylic gel. Then, the semi-dry transfer of proteins from the gel to nitrocellulose membrane was carried out. It was performed using the TransBlot (BioRad, Poland) apparatus under 15V constant voltage for 15 minutes.

#### Transfection with TAS1R genes

Genes coding the subunits of sweet taste receptors (TAS1R2/TAS1R3) were cloned into the PSF-CMV-CMV-SBFI-UB-PURO—DUAL CMV vector (Sigma Aldrich, Poland). The correctness of the cloning process was confirmed on the basis of the restriction analysis. Then, the resulted genetic construct transfected the HEK293T cell (ATCC, United States) line with the overexpression of the chimeric gustdicine response. The transfection was conducted using the lipofection method.

#### Calcium release test

Binding a ligand to the bitter/sweet taste receptor induces the release of Ca^2+^ ions from the endoplasmatic reticulum to the cytoplasmatic space. Therefore, the overactivity of bitter taste was considered to depend on a higher release of calcium, whereas bitter taste masking was considered to be associated with a decrease in the release of Ca^2+^.

The cell lines were tested in terms of the release of calcium from the endoplasmatic reticulum in the presence of an agonist of a separate receptor or in the presence of the tested cornelian cherry extract. The release of calcium was determined using the Fluo-4 Direct^™^ Calcium Assay Kit (ThermoFisher), according to the manufacturer’s protocol. Fluorescence (SpectraMax i3x, Molecular Devices, United States) was determined 1 minute after adding 5μl of the tested extract/reference material to the cells.

The following substances were used as positive control samples: 10 mM of sucralose (sweet taste receptors: TAS1R2/TAS1R3), 30 mM of chlorokin (TAS2R3 bitter taste receptors), and 30 mM of denatonium (TAS2R13 bitter taste receptor). For the purpose of positive control samples, the compounds were selected based on previous studies, according to which they are the strongest agonists [13–15].

The final result was presented as a ratio between the fluorescence growth (ΔF) of cells exposed to the *C*. *mas* extract and the intensity of fluorescence in the control sample (F_0_). The ΔF/ F_0_ value lower than 1 was considered as the deactivation of the taste receptor.

### Sensory profiling

Sensory profiling was studied in a specially designed laboratory. Each chocolate sample was randomly encoded and assessed by 10 persons. Recruited sensory assessors were in the age range of 24–40 years (6 women and 4 men); they were researchers and PhD students of Poznań University of Life Sciences, specialising in sensory studies. Before the main experiment, the assessors underwent sensory sensitivity tests, including flavour daltonism and aroma sensitivity tests. After the positive verification of the assessors, the list of sensory descriptors was developed for the tested chocolate samples and the scale with limit values for each descriptor was constructed. This part was carried out using raw materials commonly used in the preparation of chocolate, i.a. dark chocolate, milk chocolate and nuts. Between tests the assessors rinsed their mouths with table water and sniffed coffee beans to neutralise the aroma after the previous test. All three tested chocolate samples were served together on one white plate. Each assessor determined the intensity of the perceived sensory notes using a 10-point hedonic scale method (10 cm long). Each sample was served as one chocolate bar at ambient temperature. As palate cleansers mineral still water was served. The scale started with 0 (no perception) and ended with 10 (note fully peceived). The lowest value of the scale was placed on its left side. The task of each assessor was to mark the intensity of the perceived notes for all three samples on one scale. In the case of all marks, the distance was measured from the beginning of the scale and noted by the researchers. Each assessor participated in the sensory study only once. Both during the preliminary stage and the main stage of the experiment, the sensory analysis was conducted in a specially designed laboratory, where appropriate physical conditions were maintained (temperature of 21°C, relative humidity of 55–65%, air exchange of 1 cycle per 4 hours, natural lighting), and distracting factors (noise, foreign smells) were eliminated. To ensure comfort during the assessment, the tests were performed in one-person workstations (8 workstations in the laboratory). The assessors were instructed to keep chocolate samples in their mouths until they melted and to rub them on their palates. The panelists were trained that the chocolate should be kept in the mouth until it melts and rubbed on the palate and tested with the olfactory apparatus. The assessment involved properties such as flavour, aroma, colour intensity, hardness, meltability, and the general acceptance of the samples. As far as flavour is concerned, selected individual notes, such as sweet, chocolate, milk, bitter and tart flavour, were evaluated, whereas in case of aroma, overall aroma intensity, chocolate, fruit, sweet, spicy, and nut aroma was assessed.

#### Ethical statement

The participants to the sensory panel has given their oral consent to attend in the study. The results of the study presented in the paper are presented as a whole and do not show individual answers from the panelists. The results contained in the study reveal neither sensitive nor personal data of people participating in the sensory panel. Hence, a written consent declaration was not obligatory for the purpose of conducting the study or publishing its results.

The consent was confirmed by two experimentalists performing the study and was noted in laboratory notebook.

No approval from the ethics committee was needed, as this study involved no clinical trials under local law or the definition specified by the World Health Organisation.

### Total phenolic content

The total phenolic content (TPC) was conducted according to the spectrophotometric method described in Kobus-Cisowska et al. [16]. The absorbance of 725 nm was recorded by Metertech SP-830 apparatus (Taiwan). For all tested extracts the TPC was determined in triplicate. The calibration curve was based on gallic acid (Sigma Aldrich, Poland) solutions (r^2^ 0.9679). The final results were expressed as mg of gallic acid equivalents (GAE) per g of dry matter (d.m.).

### Determination of phenolic acids and flavonoids

Phenolic acids and flavonoids in the tested extracts were studied after carrying out the basic and acidic hydrolysis according to Stuper-Szablewska et al. [17]. Once the hydrolysis had been carried out, phenolic acids were extracted from the inorganic phase by means of diethyl ether. The content of phenolic acids and flavonoids was determined using a high-performance liquid chromatograph (Waters SDS 501) with a Waters 486 Tunable Absorbance Detector. The tested compounds were separated in the RP C-18 column (250 × 4 mm, 5 μm) maintained at temperature 35°C. The concentration of the compounds was determined on the basis of the internal standard, and the absorbance of λ = 320 nm and 280 nm was recorded. The eluent was a mixture of acetonitrile:2%acetic acid in water (pH = 2), and the flow rate was 0.4 ml/min. The tested compounds were identified either by comparing the retention times of the tested samples to the standards or by analysing the tested samples in relation to one of the standards. The detection level was 1 μg/g of the extract.

### Antioxidant and metal chelating activity

The antioxidant activity was performed against 2,2’-azino-bis(3-ethylbenzothiazoline-6-sulfonic acid) (ABTS) and 2,2-diphenyl-1-picrylhydrazyl (DPPH). All chemicals were purchased from Sigma Aldrich, Poznań, Poland.

The ABTS scavenging activity was determined according to Re et al. [18]. Prior to the analysis, the ABTS working solution was prepared by dissolving 0.192 g of the ABTS reagent in 50 ml of distilled water, and 16.6 mg of K_2_O_8_S_2_ (POCH, Poland) in 25 ml of distilled water. Both solutions were mixed at a volumetric ratio of 2:1 and then kept in cooling conditions (4°C) by roughly 14 h. After that time, the mixture was diluted with pure methanol (Honeywell, United Kingdom) to obtain a solution with an absorbance of 0.700 (±0.020) at a wavelength of λ = 734 nm. For the purposes of the test, 4.5 ml of the working solution was transferred to a test tube and 45μl of the tested extract was added. Then, the tube was vortexed for 5 seconds and the specimen was kept for 6 minutes in the dark. The absorbance was measured at a wavelength of 734 nm (Metertek SP-830, Taiwan) (A_p_). The control sample (A_k_) was prepared analogically, but instead of the extract, distilled water was added. The ABTS scavenging ability (A) was calculated according to Eq 1.

$$A=\frac{A_{k}-A_{p}}{A_{k}x}100$$ (1)

The calibration curve was calculated based on the trolox standard solutions (r^2^ 0.9811). The final results were expressed as μmol of trolox equivalents (TE) per d.m. All samples were tested in triplicate.

The DPPH scavenging activity was evaluated using the method of Amarowicz et al. [19]. All amount of 100 μl of the tested extract was transferred to 2.9 ml of 0.1 mM DPPH solution in a tube, and then the sample was mixed on a shaker for 3 seconds at 1500 rpm. After storing the sample in a dark room for 30 minutes, the absorbance was measured at a wavelength of 517 nm (SP-830, Metertech, Taiwan). The antioxidant activity (AA) was calculated according to Eq 2, based on the absorbance of the tested sample (E_p_), the blank (E_0_) and negative control (E_k_).

$$AA=100-\frac{\left(Ep-E0\right)}{Ek}\times 100\%$$ (2)

The blank control concerned the sample with addition of pure methanol (Honeywell, UK) instead of 1 mM DPPH methanolic solution, whereas the negative control involved the sample to which pure water was added instead of the tested extract. The calibration curve (r^2^ 0.9587) was plotted based on the trolox standard solutions, and the final results were expressed as μmol TE/d.m. Each extract was tested in triplicate.

The metal chelating ability of the chocolates were estimated according to the method described by Kobus-Cisowska et al. [16] based on the absorbance measurement (562nm) of the complex (A1) of Fe^2+^ ions not previously bonded to the extracts and 3-(2-pyridyl)-5,6-bis(4-phenyl-sulfonicacid)-1,2,4-triazine (Ferrozine, Sigma Aldrich, Poland). The final results were determined as % of the chelating activity (ChA) according to Eq 3. Each extract was tested in triplicate.

$$ChA=\frac{\left(A0-A1\right)}{A0}\times 100\%$$ (3)

The zero sample (A0) was sample with addition of pure water instead of the tested extract.

### *In vitro* intestinal digestion

The simulation of chocolate digestion process was carried out to determine the amount of polyphenols absorbed from the tested chocolate. The process was conducted in a 1L fermentation tank, maintained at a temperature of 37°C. The tested chocolate was pre-digested in gastric conditions and then the residue was subjected to the intestinal digestion. In simulated gastric conditions, pH was reduced by adding hydrochloric acid (POCH, Poland), and peristaltic movements were imitated by stirring. Then, pH was increased to 6.00 and pancreatic-intestinal extract was added. In the simulation of digestion in the large intestine, pH was increased to 8.00 and the process continued in aerobic conditions for 18 hours. The digested residue was extracted according to the method described in point 2.4 and then subjected to further analysis (points 2.5 and 2.7). The samples were encoded as follows: CHCD–digested CHC, CHFBD–digested CHFB, and CHFD–digested CHF.

### Statistical analysis

The statistical analysis was performed in Origin 2020 (Germany) Software and Microsoft Excel 2013 (United States). The descriptive statistics, one-way analysis of variance (ANOVA) and Turkey test were performed to illustrate significant differences between the tested samples (α = 0.05). As far as the results of the receptor inhibition study are concerned, statistical differences were analysed using the three-way ANOVA and two post-hoc tests, i.e. Turkey test and Scheffe test (α = 0.05). The three variables taken into account in the ANOVA were sample/receptor, reaction time and dilution. The correlation between the tested variables was illustrated using the principal component analysis (PCA). The results obtained for ten-fold diluted samples and 1-minute interaction with the tested receptors were taken into account in this model.

## Results

### Effect of *Cornus mas* on taste receptors

**Fig 1:**
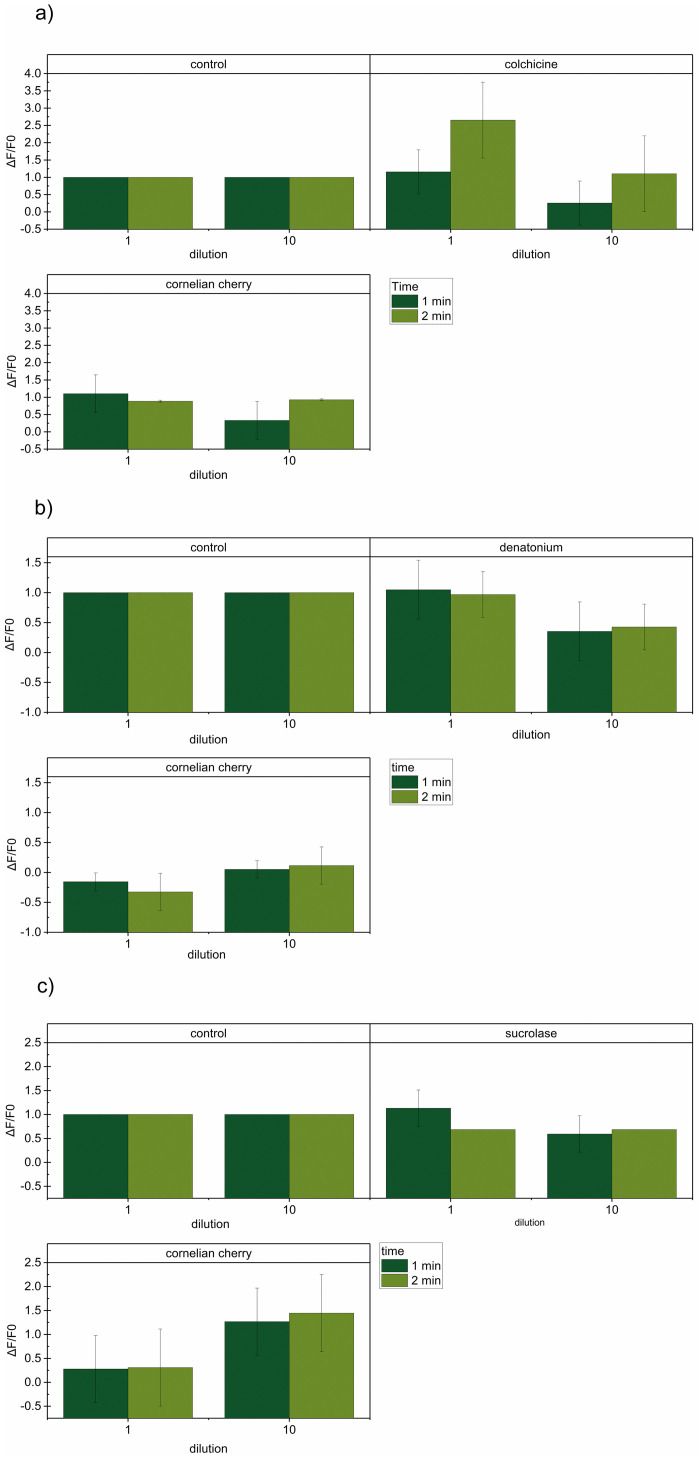
Change in fluorescence (ΔF/F0) as a response of the TAS2R3 bitter taste receptor (a) TAS2R13 bitter taste receptor (b) and TAS1R2 sweet receptor (c), induced by the tested *C*. *mas* extract and reference standards after 1 minute (dark green) and 2 minutes (light green) of interaction. N = 3.

The aim of this paper was to observe the response of taste receptors to the cornelian cherry extract. It was observed that raw cornelian cherry extract reduced the activity of the sweet taste receptor, but the 10-fold diluted extract enhanced it (Fig 1a).

Moreover, the antagonistic effect was observed in relation to the TAS2R13 bitter taste receptor for all tested cornelian cherry dilutions (Fig 1a). The strongest effect was observed after 2 minutes of interaction. For the other tested bitter taste receptor, the activity of extracts and the effect of the reference standard largely depended on time (Fig 1b). The activity of the receptor within the first minute after the consumption was rather stable, except for the BWX interaction, after which it dropped. The inhibitory effect of BWX vanished in the second minute of interaction. This clearly shows that the time of interaction between TAS2R3 and cornelian cherry active compounds is rather short.

### Sensory analysis

**Fig 2:**
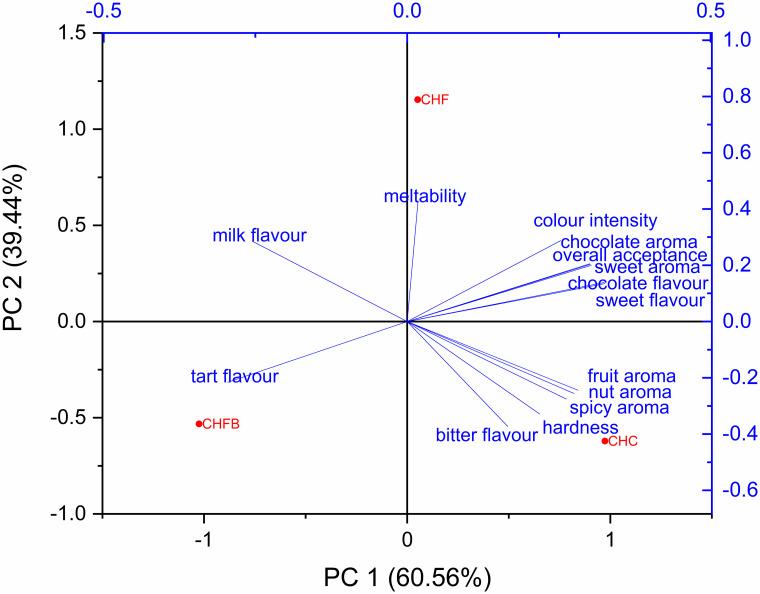
Principal scatter diagram (blue) and PCA analysis of the tested samples (red) in the centre of the figure.

Due to the fact that the taste modulating effect of *C*. *mas* fruits were observed, the study was continued with the use of fruits in sugar-free chocolate with a high cocoa content. As far as all tested notes are concerned, no significant differences were found between the tested samples (Table 1, Fig 2). Due to the fact that the control sample received the lowest number of points in respect of bitterness, it may indicate that the addition of cornelian cherry changed the overall taste perception; the bitter taste of chocolate was masked by the tart taste of fruits. The CHF variant was the sweetest sample, and it was considered to be the least bitter—3.31. The average bitterness of the CHFB sample was assessed at 5.46. The addition of cornelian cherry to dark chocolate resulted in a more significant decrease in the bitternessperceived than increase in the sweetness of chocolate.

**Table 1:** Sensory profile of the tested chocolate samples.

**SAMPLE**	**COLOUR INTENSITY**	**HARDNESS**	**MELTABILITY**	**SWEET FLAVOUR**	**CHOCOLATE FLAVOUR**	**MILK FLAVOUR**	**BITTER FLAVOUR**
**CHF**	3.90^a^ ± 2.83	5.43^a^ ± 2.41	6.72^a^ ± 1.99	6.24^a^ ± 2.55	6.85^a^ ± 1.36	5.04^a^ ± 2.12	3.31^a^ ± 1.62
**CHFB**	3.16^a^ ± 1.89	5.91^a^ ± 1.72	5.47^a^ ± 2.59	5.70^a^ ± 2.84	5.85^a^ ± 2.38	4.98^a^ ± 2.47	4.34^a^ ± 2.62
**CHC**	3.70^a^ ± 1.74	6.95^a^ ± 1.57	5.48^a^ ± 2.92	6.34^a^ ± 2.57	7.05^a^ ± 2.19	4.70^a^ ± 2.18	5.46^a^ ± 2.74
**SAMPLE**	**tart flavour**	**chocolate aroma**	**fruit aroma**	**sweet aroma**	**spicy aroma**	**nut aroma**	**general acceptance**
**CHF**	3.55^a^ ± 2.58	5.48^a^ ± 2.73	1.67^a^ ± 1.83	4.37^a^ ± 3.37	1.48^a^ ± 1.33	2.63^a^ ± 2.00	6.90^a^ ± 1.74
**CHFB**	4.22^a^ ± 2.46	4.77^a^ ± 2.99	1.69^a^ ± 1.41	3.91^a^ ± 2.88	1.73^a^ ± 1.72	2.72^a^ ± 1.71	6.06^a^ ± 2.21
**CHC**	3.59^a^ ± 1.95	5.46^a^ ± 3.12	2.09^a^ ± 2.28	4.36^a^ ± 2.67	3.20^a^ ± 2.65	3.70^a^ ± 2.78	6.93^a^ ± 1.61

Results given as mean ± standard deviation (N = 10), CHF–chocolate with cornelian cherry fruits, CHFB–chocolate with cornelian cherry fruits and *B*. *coagulans*, CHC–control chocolate, lowercase letters show significant differences between the samples (α = 0.05).

As far as the aroma is concerned, the addition of cornelian cherry had no negative effect on the intensity of general aroma and the intensity of two key notes (Fig 2). The highest note for chocolate aroma was resulted for CHF.

The CHC variant was the smoothest and the hardest (7.56 and 6.95, respectively) (Fig 2), whereas the CHF sample was the most meltable (6.72) but the least hard (5.43). The CHFB variant received the lowest mark for meltability, which leads to the conclusion that the addition of cornelian cherry negatively affects the meltability of the chocolate layer and might induce the appearance of fat or sugar blooms.

All tested chocolate samples received low marks for their colour (Fig 2). The CHFB variant was the darkest, but differences in colour were not significant. As a result, it was observed that the addition of cornelian cherry to chocolate does not affect its colour.

### Total polyphenol content

**Fig 3:**
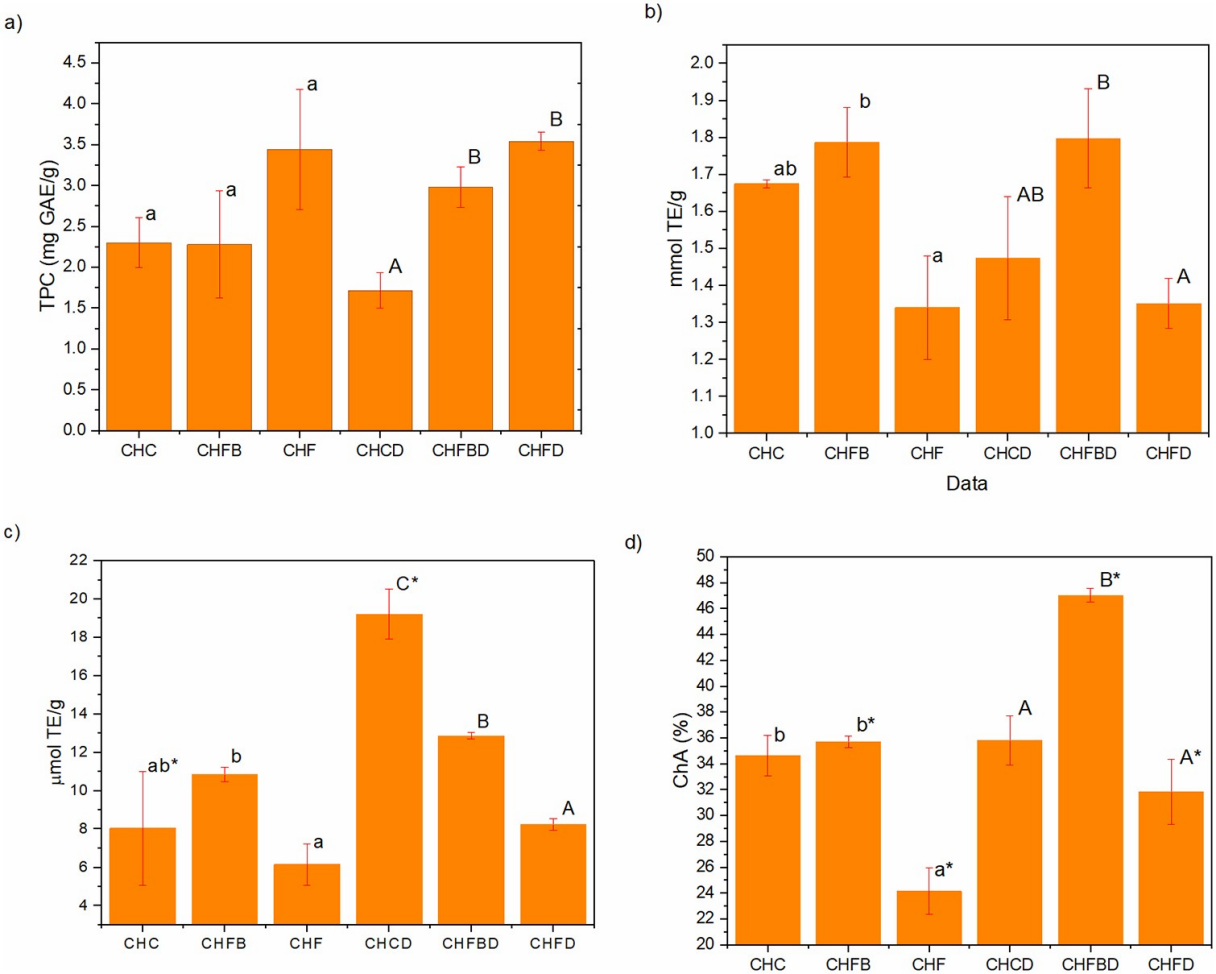
Total phenolic content (a), antioxidant activity against ABTS (b), antioxidant activity against DPPH (c) and chelating ability (d) of the tested chocolate samples before and after the in vitro digestion process. N = 3. Lowercase a-b letters indicate significant differences between indigested samples. Capital letters A-C represent significant differences between digested samples. * -significant difference between indigested and digested samples of the same chocolate variant. P(0.002)<0.05.

The highest TPC was observed in the CHF sample (3.44 mg GAE/g of the product), whereas the lowest value was recorded for the CHFB (2.28 mg GAE/g) (Fig 3a).

### Polyphenolic acids and flavonoid content

As many as twenty compounds were detected in the tested chocolate samples (Table 2). The prevalent phenolic acids were 2,5-dihrydoxybenzoic acid, 4-hydroxybenzoic acid, and chlorogenic acid, the content of which was significantly higher than that of the other two acids. Flavonoids included naringin (1403.0–1695.0 μg/g d.m.), catechin (1359.0–1422.0 μg/g d.m.), and apigenin (658.0–705.0 μg/g d.m.).

**Table 2:** Content (μg/g d.m.) of phenolic acids and flavonoids in the tested samples.

**SAMPLE**	**GALLIC ACID**	**2,5-DIHYDROXYBENZOIC ACID**	**4-HYDROXYBENZOIC ACID**	**CAFFEIC ACID**	**SYRINGIC ACID**	*P*-COUMARIC ACID	**FERULIC ACID**	**CHLOROGENIC ACID**	**SYNAPIC ACID**	*T*-CINNAMIC ACID
**BW**	17,081.6^eC^ ±180.8	1,945.4^cC^ ±137.8	565.3^abD^±59.2	177.7^aB^±6.0	419.7^aC^±20.2	713.9^bC^±21.9	264.5^aB^±21.1	538.5^abB^±24.9	248.0^aC^±7.0	424.5^aC^±28.5
**CHF**	30.5^aB^ ±1.8	4.4^aA^±0.7	6.9^aB^±0.9	1.7^aA^ ±0.4	16.0^a A^±1.3	67.0^aB^ ±2.7	39.0^aA^ ±2.1	569.5^bB^ ±8.0	1.0^aA^ ±0.3	59.0^aA^ ±2.6
**CHFB**	31.6^aB^ ±1.9	49.0^aB^±2.3	18.0^aC^±1.4	243.0^aC^ ±5.2	26.0^aB^±1.7	59.0^aA^ ±2.6	43.0^aA^ ±2.2	615.2^bC^ ±8.3	16.0^aB^±1.3	68.0^aB^±2.7
**CHC**	1.2^aA^±0.4	2.3^aA^±0.5	2.1^aA^±0.5	1.3^aA^ ±0.4	15.2^aA^ ±1.3	67.3^aB^ ±2.7	39.0^aA^ ±2.1	218.4^bA^ ±4.9	0.2^aA^ ±0.1	61.4^aA^ ±2.6
**SAMPLE**	**vanillic acid**	**salicylic acid**	**naringenin**	**vitexin**	**rutin**	**quercetin**	**apigenin**	**kaempferol**	**luteolin**	**catechin**
**BW**	121.4^aA^ ±6.2	393.5^aC^ ±34.2	45,863.1^fD^ ±899.9	4,445.0^dC^±131.9	35,604.2^efC^±295.7	760.7^bC^±29.0	26.0^aA^±5.3	31.9^aA^±4.1	18.6^aA^±1.4	26,121.2^eC^±178.0
**CHF**	269.0^aBC^ ±5.5	1.2^aA^ ±0.4	1,452.0^cA^ ±12.7	165.0^aA^ ±4.3	96.0^aB^ ±3.3	236.0^aA^ ±5.1	658.0^cB^ ±8.6	346.0^aB^ ±6.2	52.0^aC^ ±2.4	1,365.0^cA^ ±2.4
**CHFB**	302.0^aC^ ±5.8	10.9^aB^ ±1.1	1,695.0^cB^ ±13.7	186.0^aB^ ±4.6	87.0^aB^ ±3.1	398.0^aB^ ±6.6	705.0^cC^ ±8.8	386.0^aC^ ±6.5	45.0^aB^ ±2.2	1,422.0^cB^ ±2.2
**CHC**	259.0^aB^ ±5.4	0.9^aA^ ±0.3	1,403.0^cA^ ±12.5	161.0^aA^ ±4.2	53.0^aA^ ±2.4	200.5^aA^ ±4.7	668.5^cB^ ±8.6	337.4^aB^ ±6.1	62.3^aD^ ±2.6	1,359.0^cA^ ±2.6

N = 3, BW–cornelian cherry fruits; CHF–chocolate with cornelian cherry fruits, CHFB–chocolate with cornelian cherry and *B*. *coagulans*, CHC–control chocolate, NE–not examined lowercase letters show significant differences between the tested compounds (α = 0.05), superscript letters present significant differences between the tested samples (α = 0.05).

The control samples were characterised by the poorest number of the tested compounds. A higher content was observed for other tested chocolate samples. Both CHF and CHFB variants were rich in 2,5-dihydroxybenzoic, 4-hydroxybenzoic, caffeic, and sinapic acids, as well as in rutin (Table 2).

Chocolate with the addition of probiotics had a significantly higher content of naringin and quercetin; the content of 2,5-dihydroxybenzoic acid in this chocolate variant was over ten-times higher than in CHF. The CHFB variant had over 12-times higher content of salicylic acid, and over 180-times higher content of caffeic acid, than the control sample.

### Antioxidant and metal chelating activity

It was observed that the CHFB variant demonstrated the strongest, and the CHP sample the poorest, inhibiting activity on ABTS (1.786 mmol TE/g and 1.340 mmol TE/g, respectively) (Fig 3b). After the digestion process, the tested samples had similar radical scavenging ability (1.797 and 1.350 mmol TE/g, respectively), which may indicate that it might have no negative effect on their functional properties.

Similar activities were observed against both and ABTS. The highest scavenging ability had CHFB (10.848 μmol TE/g) and the lowest—for CHF (6.150 μmol TE/g). Surprisingly, after carrying out the digestion simulation, the control sample demonstrated the highest activity against DPPH, which was over twice higher than before the digestion process (Fig 3c). Other digested samples resulted in values comparable to those recorded for indigested chocolate.

The chelating ability of CHFB (35.7%) did not differ significantly from the control sample; in the case of CHF, the chelating ability was approx. 30% lower (Fig 3d). As far as the digested samples are concerned, both chocolate variants with *C*. *mas* saw a significant increase in their chelating. The highest chelating ability had CHFBD (47.0%), while between CHC and CHCD no significant difference was noted.

### Statistical analysis

**Fig 4:**
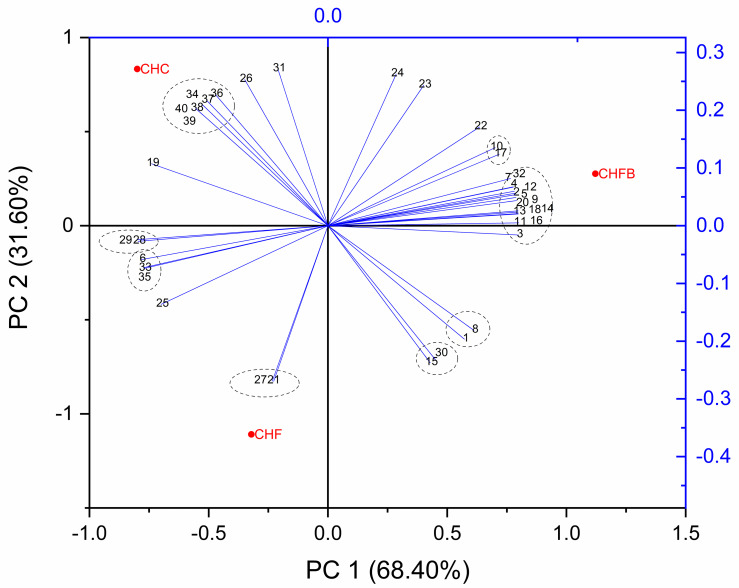
Principal component analysis of the tested chocolate sample (red) and variables (blue). 1- gallic acid; 2–2.5-dihydroxybenzoic acid; 3–4-hydroxybenzoic acid; 4- caffeic acid;5—syringic acid; 6—p-coumaric acid; 7—ferulic acid; 8—chlorogenic acid; 9—sinapic acid; 10—t-cinnamic acid; 11—vanilic acid; 12—salicylic acid; 13 –naringenin; 14 –vitexin; 15 –rutin; 16 –quercetin; 17 –apigenin; 18 –kaempferol; 19 –luteolin; 20 –catechin; 21 –TPC; 22 –DPPH; 23—ABTS; 24 –ChA; 25—colour intensity; 26 –hardness; 27 –meltability; 28—sweet flavour; 29—chocolate flavour; 30—milk flavour; 31—bitter flavour; 32—tart flavour; 33—chocolate aroma; 34—fruit aroma; 35—sweet aroma; 36—spicy aroma; 37—nut aroma; 38—deactivation of TAS2R3 receptor; 39 –deactivation of TAS2R13; 40 –dectivation of TAS1R2.

All three tested chocolate variants differed significantly (Fig 4). The results of the CHF sample strongly depended on its TPC and melatbility, whereas the results obtained for the chocolate variant with *B*. *coagulans* (CHFB) were largely influenced by the content of individual phenolic acids and flavonoids. What is more, the CHFB sample was located in remote position in positive ranges of both statistical factors, contrary to the CHF. The catechin content can be found in the centre of PCA plot, which leads to the conclusion that this phytocompound had the highest effect on the studied samples and variables.

In the TAS2R and TAS1R study, the differences between the standards and the cornelian cherry extract were not significant according to the post-hoc Turkey test (p = 1.00). Moreover, no significant differences were found between the sample dilutions used in the tests and those used for the time of interaction. These findings were confirmed based on the post-hoc test (S2–S7, S9–S14, S16–S21 Tables). However, to draw stronger conclusions, a larger number of variables and samples would have to be studied. The deactivation of the TAS2R and TAS1R receptors by *C*. *mas* strongly correlated with the spicy and nut aroma of the tested chocolate samples (Fig 4). A close correlation was also observed between the bitter flavour, fruit aroma and luteolin content. On the other hand, these variables were the most important factors determining the properties of the CHC variant, i.e. chocolate without the addition of cornelian cherry. This indicates that the bitterness masking effect of cornelian cherry may be hardly noticeable in the final product.

## Discussion

The addition of natural antioxidants to the fruit matrix results in a specific, characteristic flavour and aroma, whereas raw materials rich in polyphenols may give tart and bitter flavours. Lončarević et al. [20] showed that the addition of blackberry juice had an indirect effect on the final taste of white chocolate due to induced rheological changes. These alterations mainly affected the texture of the product; changes in taste were more difficult to observe. However, the addition of freeze-dried fruits of *Garcinia mangostana* Linn to dark chocolate did not affect the texture and sensory profile of the product, and only an increase in the polyphenol content was observed [21]. According to many studies [22–24], changes in the perception of flavour induced by plant additives to the chocolate matrix depend on numerous factors. The structure and texture of chocolate are also important. Chocolate with a higher fat content has a more intense taste than low-fat one. The content of individual polyphenols in the additive used is also important due to the fact that the chemical structure of phenolic compounds plays a key role in the perception of flavour [25].

The sweet taste receptor consists of two cooperating subunits, TAS1R3 and TAS1R3, which interact with various chemical substances, including sugars (glucose, sucrose, fructose, and sugar alcohols), D-amino acids (D-tryptophan and D-phenylalanine), glycosides, and artificial sweeteners (sucralose, aspartame, neotame, saccharin Na, acesulfame K, and cyclamate) [26]. The presence of sugars in cornelian cherry has been confirmed in many studies, which have indicated that glucose is the dominant sugar (43–61% of total sugars– 11.2–14.9 g/100 g) [12, 27, 28].

The HPLC analysis showed significant differences between cornelian cherry fruits and the chocolate samples (Table 1). The content of naringin and gallic acid in the tested chocolate samples was approx. 500- and 27-times lower than in the fruit extract. On the other hand, the tested chocolate variants had a higher content of vanillic acid, kaempferol, and apigenin (2.5-, 27- and 12-times higher, respectively).

The total polyphenol content in cornelian cherry was 5.40 mg GAE/g dm for the aqueous extract and even twice as high for the water-ethanol extract [12]. Cerit et al. [29] tested the functional potential of the *C*. *mas* fruit content of 2% in white chocolate. The TPC estimated in the above-mentioned study was 0.16 mg GAE/g dm, and it was not detected in the control sample. The addition of cornelian cherry in the study led to a stronger increase in the tested parameter due to the synergic effect of dark cocoa ingredients.

In addition to flavonoids, sinapic acid, ferulic acid, and chlorogenic acid were also detected in the tested chocolate samples and *Cornus mas* extracts. These compounds have been reported to act as bitter off-taste inhibitors [30].

According to Roland et al. [31], the structure of phenolic compounds determined the strength of interaction with the TAS2R receptor, especially the presence of sugar moieties at position 3 and the location of the methoxy group. The results of that study showed that the activating effect of *C*. *mas* on the TAS2R receptor can result from apigenin, catechin, kaempferol, and naringin. Although the bitterness of naringin is similar to that of caffeine, the sensory panel considered sweet flavour as the most intense note after the addition of naringin to a 5% sucralose solution [32]. Due to its specific, partially planar structure, naringin is a very strong ligand interacting with various macromolecules, including both protein receptors and double-stranded DNA as well as oligonucleotides [33, 34]. Moreover, the affinity of phenolic compounds differs significantly between the TAS2R receptors, e.g. the lowest concentration of kaempferol activating TAS2R14 and TAS2R39 is 8 and 0.5 μM, respectively [35]. Di Pizio et al. [36] noted that TAS2Rs varied in the number of agonists as well as chemical interactions.

Biological functions of polyphenols depend on their conversion in the gastrointestinal tract and the chemical structure of the metabolites obtained. Likewise, gastric bioconversion affects the antioxidant activity. It has been demonstrated that the digestion process has a significant effect on wine and grape polyphenol contents [37]. Phenolic acids were found to be the least labile in the digestion process; they were associated with the antioxidant activity, which was not significantly reduced in digestion conditions. A correlation between the TPC and the antioxidant activity was observed as the latter was related to the digestion stage. Tarko et al. [38] also observed that the antioxidant activity depended on the raw material and the composition of individual polyphenolic compounds. It was noted that the compounds obtained after the digestion of chokeberries, pears and bananas had a lower antioxidant potential than undigested fruits; opposite results were however achieved in the case of apples and pears. All digested samples had a lower TPC than the undigested ones (fresh fruits). The authors of the above-mentioned study concluded that polyphenols were subjected to hydrolysis during the digestion process, especially quercetin and cyaniding glycosides. It is possible that conjugation with glucuronic acid or sulphating is the final stage of phenolic acid conversion, while methylation is the final phase of the conversion of flavonoids. The first mechanism may indicate the hydrophobic properties of phenolic acid glycosides and their electron density, as a result of which their antioxidant properties differ from those of aglycones [39].

Chocolate with the addition of probiotic bacteria *B*. *coagulans* (CHFB) was the richest source of naringin and quercetin. The effect of probiotic bacteria on polyphenol metabolism has been confirmed in numerous studies [40–42]. It was demonstrated that the presence of *L*. *plantarum* CLC 17 at the final stage of the digestion process (colon) resulted in a higher amount of phenolic metabolites, such as benzoic acid analogues, which probably resulted from the degradation of procyanidin polymers [40]. *Bacillus coagulans* has been the subject of many recent studies [11, 43, 44] due to its high tolerance to extreme conditions and probiotic properties. The safety of its used in food products has also been confirmed [41]. In our previous experiment [11] with chocolate enriched with *B*. *coagulans*, the addition of bacteria did not significantly affect the content of the tested polyphenols, no change in the colour and consistency of chocolate was observed.

## Conclusions

Phytocompounds in cornelian cherry may modulate the activity of bitter taste receptors. The activity of cornelian cherry extracts on the TAS1R and TAS2R receptors depended on time. The longest interaction between the extract ingredients and the receptors had a negative impact on the perception of bitter taste and a positive impact on the expression of sweet taste receptors. Bitter taste modulating properties may also be observed in the food matrix (i.e. chocolate), in case of which the sensory analysis showed that the overall expression perceived for chocolate enriched with *Cornus mas* reduced its bitterness rather than increased its sweetness. The chocolate samples prepared with the addition of cornelian cherry fruits resulted in a high phenolic acid and flavonoid content and acted as good radical quenchers. A 5-percent addition of cornelian cherry fruits was found to be optimal for the mass production of chocolate. Such an addition may act as a bitter taste masking agent and increase the antioxidant capacity. After the *in vitro* digestion process, chocolate enriched with probiotics (CHFB) showed no significant changes in its qualitative and quantitative content of phenolic compounds. Moreover, it was observed that the DPPH scavenging ability and the chelating activity of the sample containing *C*. *mas* and *B*. *coagulans* increased significantly. However, the addition of probiotics reduced the perception of the tested sensory notes. The chocolate prepared for the purpose of this study may be an attractive snack for consumers who are on a low-sugar diet or for consumers seeking new functional products.

## Supporting information

S1 Table ANOVA analysis of the TAS1R2 interaction. DF–degrees of freedom.
(DOCX)

S2 Table Turkey test of comparisons between tested samples against TAS1R2 receptor. SEM–standard error of the mean; Prob–Probability; Sig–Significance (0 –no significance; 1 –significance confirmed).
(DOCX)

S3 Table Turkey test of significant differences between tested interaction times (1 and 2 min) against TAS1R2 receptor. SEM–standard error of the mean; Prob–Probability; Sig–Significance (0 –no significance; 1 –significance confirmed).
(DOCX)

S4 Table Turkey test of significant differences between tested dilutions (1 and 10x) against TAS1R2 receptor. SEM–standard error of the mean; Prob–Probability; Sig–Significance (0 –no significance; 1 –significance confirmed).
(DOCX)

S5 Table Scheffe test of comparisons between tested samples against TAS1R2 receptor. SEM–standard error of the mean; Prob–Probability; Sig–Significance (0 –no significance; 1 –significance confirmed).
(DOCX)

S6 Table Scheffe test of significant differences between tested interaction times (1 and 2 min) against TAS1R2 receptor. SEM–standard error of the mean; Prob–Probability; Sig–Significance (0 –no significance; 1 –significance confirmed).
(DOCX)

S7 Table Scheffe test of significant differences between tested dilutions (1 and 10x) against TAS1R2 receptor. SEM–standard error of the mean; Prob–Probability; Sig–Significance (0 –no significance; 1 –significance confirmed).
(DOCX)

S8 Table ANOVA analysis of the TAS2R3 interaction. DF–degrees of freedom.
(DOCX)

S9 Table Turkey test of comparisons between tested samples against TAS2R3 receptor. SEM–standard error of the mean; Prob–Probability; Sig–Significance (0 –no significance; 1 –significance confirmed).
(DOCX)

S10 Table Turkey test of significant differences between tested interaction times (1 and 2 min) against TAS2R3 receptor. SEM–standard error of the mean; Prob–Probability; Sig–Significance (0 –no significance; 1 –significance confirmed).
(DOCX)

S11 Table Turkey test of significant differences between tested dilutions (1 and 10x) against TAS2R3 receptor. SEM–standard error of the mean; Prob–Probability; Sig–Significance (0 –no significance; 1 –significance confirmed).
(DOCX)

S12 Table Scheffe test of comparisons between tested samples against TAS2R3 receptor. SEM–standard error of the mean; Prob–Probability; Sig–Significance (0 –no significance; 1 –significance confirmed).
(DOCX)

S13 Table Scheffe test of significant differences between tested interaction times (1 and 2 min) against TAS2R3 receptor. SEM–standard error of the mean; Prob–Probability; Sig–Significance (0 –no significance; 1 –significance confirmed).
(DOCX)

S14 Table Scheffe test of significant differences between tested tested dilutions (1 and 10x) against TAS2R3 receptor. SEM–standard error of the mean; Prob–Probability; Sig–Significance (0 –no significance; 1 –significance confirmed).
(DOCX)

S15 Table ANOVA analysis of the TAS2R13 interaction. DF–degrees of freedom.
(DOCX)

S16 Table Turkey test of comparisons between tested samples against TAS2R13 receptor. SEM–standard error of the mean; Prob–Probability; Sig–Significance (0 –no significance; 1 –significance confirmed).
(DOCX)

S17 Table Turkey test of significant differences between tested interaction times (1 and 2 min) against TAS2R13 receptor. SEM–standard error of the mean; Prob–Probability; Sig–Significance (0 –no significance; 1 –significance confirmed).
(DOCX)

S18 Table Turkey test of significant differences between tested tested dilutions (1 and 10x) against TAS2R13 receptor. SEM–standard error of the mean; Prob–Probability; Sig–Significance (0 –no significance; 1 –significance confirmed).
(DOCX)

S19 Table Scheffe test of comparisons between tested samples against TAS2R13 receptor. SEM–standard error of the mean; Prob–Probability; Sig–Significance (0 –no significance; 1 –significance confirmed).
(DOCX)

S20 Table Scheffe test of significant differences between tested interaction times (1 and 2 min) against TAS2R13 receptor. SEM–standard error of the mean; Prob–Probability; Sig–Significance (0 –no significance; 1 –significance confirmed).
(DOCX)

S21 Table Scheffe test of significant differences between tested tested dilutions (1 and 10x) against TAS2R13 receptor. SEM–standard error of the mean; Prob–Probability; Sig–Significance (0 –no significance; 1 –significance confirmed).
(DOCX)
