# Supplementary material for: Can cornelian cherry mask bitter taste of probiotic chocolate? Human TAS2R receptors and a sensory study with comprehensive characterisation of new functional product
Source: PLoS One. 2021 Feb 8;16(2):e0243871. doi: 10.1371/journal.pone.0243871 (PMC7869990; doi:10.1371/journal.pone.0243871)
Supplement: S1 Table — DF–degrees of freedom. (DOCX) [file pone.0243871.s001.docx]

**S1 Table. ANOVA analysis of the TAS1R2 interaction**

| variable(s) | DF | Sum of Squares | Mean Square | F Value | P Value |
| --- | --- | --- | --- | --- | --- |
| sample | 2 | 0.11231 | 0.05616 | 0 | 1 |
| time | 1 | 0.00172 | 0.00172 | 0 | 1 |
| dilution | 1 | 0.21048 | 0.21048 | 0 | 1 |
| sample * time | 2 | 0.04031 | 0.02016 | 0 | 1 |
| sample * dilution | 2 | 0.9935 | 0.49675 | 0 | 1 |
| time * dilution | 1 | 0.03921 | 0.03921 | 0 | 1 |
| sample * time * dilution | 2 | 0.03867 | 0.01933 | 0 | 1 |
| Model | 11 | 1.43621 | 0.13056 | 0 | 1 |
| Error | 0 | 1.77636E-15 | -- | 0 | 0 |
| Corrected Total | 11 | 1.43621 | 0 | 0 | 0 |

DF – degrees of frredom
