# Supplementary material for: Can cornelian cherry mask bitter taste of probiotic chocolate? Human TAS2R receptors and a sensory study with comprehensive characterisation of new functional product
Source: PLoS One. 2021 Feb 8;16(2):e0243871. doi: 10.1371/journal.pone.0243871 (PMC7869990; doi:10.1371/journal.pone.0243871)
Supplement: S2 Table — SEM–standard error of the mean; Prob–Probability; Sig–Significance (0 –no significance; 1 –significance confirmed). (DOCX) [file pone.0243871.s002.docx]

**S2 Table. Turkey test of comparisons between tested samples against TAS1R2 receptor.**

| sample vs sample | MeanDiff | SEM | q Value | Prob | Alpha | Sig |
| --- | --- | --- | --- | --- | --- | --- |
| control sucrolase | 0.22597 | 9.21803E-16 | 3.46682E14 | 1 | 0.05 | 0 |
| control cornelian cherry | 0.17478 | 9.21803E-16 | 2.6815E14 | 1 | 0.05 | 0 |
| sucrolase cornelian cherry | -0.05119 | 9.21803E-16 | -7.85328E13 | 1 | 0.05 | 0 |

SEM – standard error of the mean; Prob – Probability; Sig – Significance (0 – no significance; 1 – significance confirmed).
