# Supplementary material for: Can cornelian cherry mask bitter taste of probiotic chocolate? Human TAS2R receptors and a sensory study with comprehensive characterisation of new functional product
Source: PLoS One. 2021 Feb 8;16(2):e0243871. doi: 10.1371/journal.pone.0243871 (PMC7869990; doi:10.1371/journal.pone.0243871)
Supplement: S5 Table — SEM–standard error of the mean; Prob–Probability; Sig–Significance (0 –no significance; 1 –significance confirmed). (DOCX) [file pone.0243871.s005.docx]

**S5 Table. Scheffe test of comparisons between tested samples against TAS1R2 receptor.**

| sample vs. sample | MeanDiff | SEM | F Value | Prob | Alpha | Sig |
| --- | --- | --- | --- | --- | --- | --- |
| control sucrolase | 0.22597 | 9.21803E-16 | 2.45142E14 | 1 | 0.05 | 0 |
| control cornelian cherry | 0.17478 | 9.21803E-16 | 1.8961E14 | 1 | 0.05 | 0 |
| sucrolase cornelian cherry | -0.05119 | 9.21803E-16 | -5.55311E13 | 1 | 0.05 | 0 |

SEM – standard error of the mean; Prob – Probability; Sig – Significance (0 – no significance; 1 – significance confirmed).
