# Supplementary material for: Can cornelian cherry mask bitter taste of probiotic chocolate? Human TAS2R receptors and a sensory study with comprehensive characterisation of new functional product
Source: PLoS One. 2021 Feb 8;16(2):e0243871. doi: 10.1371/journal.pone.0243871 (PMC7869990; doi:10.1371/journal.pone.0243871)
Supplement: S8 Table — DF–degrees of freedom. (DOCX) [file pone.0243871.s008.docx]

**S8 Table. ANOVA analysis of the TAS2R3 interaction**

| variable(s) | DF | Sum of Squares | Mean Square | F Value | P Value |
| --- | --- | --- | --- | --- | --- |
| sample | 2 | 0.46857 | 0.23428 | 0 | 1 |
| time | 1 | 0.61679 | 0.61679 | 0 | 1 |
| dilution | 1 | 0.84508 | 0.84508 | 0 | 1 |
| sample * time | 2 | 0.79136 | 0.39568 | 0 | 1 |
| sample * dilution | 2 | 0.79377 | 0.39689 | 0 | 1 |
| time * dilution | 1 | 0.00221 | 0.00221 | 0 | 1 |
| sample * time * dilution | 2 | 0.26792 | 0.13396 | 0 | 1 |
| Model | 11 | 3.7857 | 0.34415 | 0 | 1 |
| Error | 0 | 3.55271E-15 | -- | 0 | 0 |
| Corrected Total | 11 | 3.7857 | 0 | 0 | 0 |

DF – degrees of freedom
