# Supplementary material for: Can cornelian cherry mask bitter taste of probiotic chocolate? Human TAS2R receptors and a sensory study with comprehensive characterisation of new functional product
Source: PLoS One. 2021 Feb 8;16(2):e0243871. doi: 10.1371/journal.pone.0243871 (PMC7869990; doi:10.1371/journal.pone.0243871)
Supplement: S15 Table — DF–degrees of freedom. (DOCX) [file pone.0243871.s015.docx]

**S 15 Table. ANOVA analysis of the TAS2R13 interaction**

| variable(s) | DF | Sum of Squares | Mean Square | F Value | P Value |
| --- | --- | --- | --- | --- | --- |
| sample | 2 | 2.47696 | 1.23848 | 0 | 1 |
| time | 1 | 0.00112 | 0.00112 | 0 | 1 |
| dilution | 1 | 0.02924 | 0.02924 | 0 | 1 |
| sample * time | 2 | 0.0018 | 9.01413E-4 | 0 | 1 |
| sample * dilution | 2 | 0.4578 | 0.2289 | 0 | 1 |
| time * dilution | 1 | 0.01266 | 0.01266 | 0 | 1 |
| sample * time * dilution | 2 | 0.00715 | 0.00357 | 0 | 1 |
| Model | 11 | 2.98672 | 0.27152 | 0 | 1 |
| Error | 0 | -1.77636E-15 | -- | 0 | 0 |
| Corrected Total | 11 | 2.98672 | 0 | 0 | 0 |

DF – degrees of freedom
