# Supplementary material for: Can cornelian cherry mask bitter taste of probiotic chocolate? Human TAS2R receptors and a sensory study with comprehensive characterisation of new functional product
Source: PLoS One. 2021 Feb 8;16(2):e0243871. doi: 10.1371/journal.pone.0243871 (PMC7869990; doi:10.1371/journal.pone.0243871)
Supplement: S17 Table — SEM–standard error of the mean; Prob–Probability; Sig–Significance (0 –no significance; 1 –significance confirmed). (DOCX) [file pone.0243871.s017.docx]

**S 17 Table. Turkey test of significant differences between tested interaction times (1 and 2 min) against TAS2R13 receptor.**

| MeanDiff | SEM | q Value | Prob | Alpha | Sig |
| --- | --- | --- | --- | --- | --- |
| 0.01931 | 7.55548E-16 | 3.61507E13 | 1 | 0.05 | 0 |

SEM – standard error of the mean; Prob – Probability; Sig – Significance (0 – no significance; 1 – significance confirmed).
