# Supplementary material for: Can cornelian cherry mask bitter taste of probiotic chocolate? Human TAS2R receptors and a sensory study with comprehensive characterisation of new functional product
Source: PLoS One. 2021 Feb 8;16(2):e0243871. doi: 10.1371/journal.pone.0243871 (PMC7869990; doi:10.1371/journal.pone.0243871)
Supplement: S19 Table — SEM–standard error of the mean; Prob–Probability; Sig–Significance (0 –no significance; 1 –significance confirmed). (DOCX) [file pone.0243871.s019.docx]

**S19 Table. Scheffe test of comparisons between tested samples against TAS2R13 receptor.**

| sample vs. sample | MeanDiff | SEM | F Value | Prob | Alpha | Sig |
| --- | --- | --- | --- | --- | --- | --- |
| control denatonium | 0.30097 | 9.25353E-16 | 3.25248E14 | 1 | 0.05 | 0 |
| control cornelian cherry | 1.07834 | 9.25353E-16 | 1.16533E15 | 1 | 0.05 | 0 |
| denatonium cornelian cherry | 0.77737 | 9.25353E-16 | 8.40084E14 | 1 | 0.05 | 0 |

SEM – standard error of the mean; Prob – Probability; Sig – Significance (0 – no significance; 1 – significance confirmed).
